# A theory for why the spaceflight-associated neuro-ocular syndrome develops

**DOI:** 10.1152/japplphysiol.00854.2021

**Published:** 2022-02-24

**Authors:** Jay C. Buckey, Mimi Lan, Scott D. Phillips, Veronique Archambault-Leger, Abigail M. Fellows

**Affiliations:** ^1^Geisel School of Medicine, Dartmouth College, Hanover, New Hampshire; ^2^Thayer School of Engineering, Dartmouth College, Hanover, New Hampshire; ^3^Creare LLC, Hanover, New Hampshire

**Keywords:** hydrostatic gradients, microgravity, numerical model, spaceflight-associated neuro-ocular syndrome

The spaceflight-associated neuro-ocular syndrome (SANS) occurs in some long-duration astronauts and includes ocular changes such as globe flattening and optic disk edema (1–3). This syndrome has defied an easy explanation. Because of the globe flattening, some suggested SANS resembled idiopathic intracranial hypertension. This, combined with the distended appearance of jugular veins in space, led to the hypothesis that intracranial pressure (ICP) was elevated in space possibly due to increased venous pressures in the head.

This hypothesis had problems. Elevated head venous pressures should increase not only ICP but also intraocular pressure (IOP). Consistently increased IOP in space compared with supine measurements has not been seen (4). Also, starting with the pioneering measurements of peripheral venous pressure by Kirsch et al. (5) on the Spacelab 1 flight and followed by the measurements of central venous pressure (CVP) on the Spacelab Life Sciences-1, Spacelab Life Sciences-2, and Deutsche-2 missions, venous pressures have been at or below supine levels in weightlessness (5–9). No plausible theory exists to explain how internal jugular venous vein (IJV) pressure could be elevated above supine levels, when peripheral venous pressure, CVP, and IOP are not.

The reduced venous pressures could be explained by the removal of tissue compressive forces (9, 10). These are forces on the outside of blood vessels due to the weight of the tissue produced by gravity. The removal of these forces could help explain the seemingly paradoxical CVP results, which included a reduction in CVP below supine levels combined with an increase in stroke volume. Ordinarily, a decrease in CVP would be expected to decrease stroke volume.

Eliminating tissue compressive forces is not possible on Earth but can be simulated using numerical modeling. We developed a multicompartment, lumped-parameter, numerical model of the cranial circulation to develop hypotheses for SANS (10). The model has a circulatory submodel, a CSF submodel, and an aqueous humor submodel implemented using MATLAB Simscape Fluids (MathWorks, Natick, MA) (11). It incorporates tissue-weight generated compressive forces exerted on vessels, venous and arterial hydrostatic gradients, and the removal of those elements in microgravity. The model showed CVP and ICP fell in weightlessness relative to supine. IOP in weightlessness was also reduced relative to supine, and this was greater in those with greater tissue weight (12).

To determine whether the relationship between IOP and body weight had experimental support, we reviewed published studies for the relationship between IOP and body weight (13). This review showed a positive relationship between body weight and IOP and found that reducing body weight (for example, through weight loss surgery) was associated with reducing IOP. To determine if body weight is associated with SANS, we analyzed data from the Longitudinal Study of Astronaut Health. This showed a strong and significant positive relationship between the risk of developing SANS and preflight body weight (14). These findings seemed to make SANS harder to explain. If removing tissue weight reduces venous pressures, then those with the greatest weights might be expected to see the greatest reductions in venous pressure in space compared with supine values. These reductions in venous pressures would seem to make SANS less likely to develop because reduced venous pressures might be expected to lead to reduced ICP.

To reconcile these seemingly disparate findings we did a theoretical analysis of the hydrostatic pressure changes likely to occur in the head with weightlessness. Figure 1*A* describes the possible situation with hydrostatic pressures in the supine position on Earth. For this analysis, we considered that the most important pressures were the IOP at the back of the eye (IOP_back of eye_) and the ICP at the back of the eye (ICP_back of eye_). The difference between these two pressures is the transmural pressure across the back of the eye (IOP_back of eye_–ICP_back of eye_). A reduced transmural pressure promotes eye shortening, whereas an increase would promote lengthening. The shaded arrows represent the hydrostatic gradients within the eye and the head, respectively. An important observation is that supine ICP_back of eye_ is less than the mean ICP in the head because of hydrostatic gradients.

**Figure 1.**
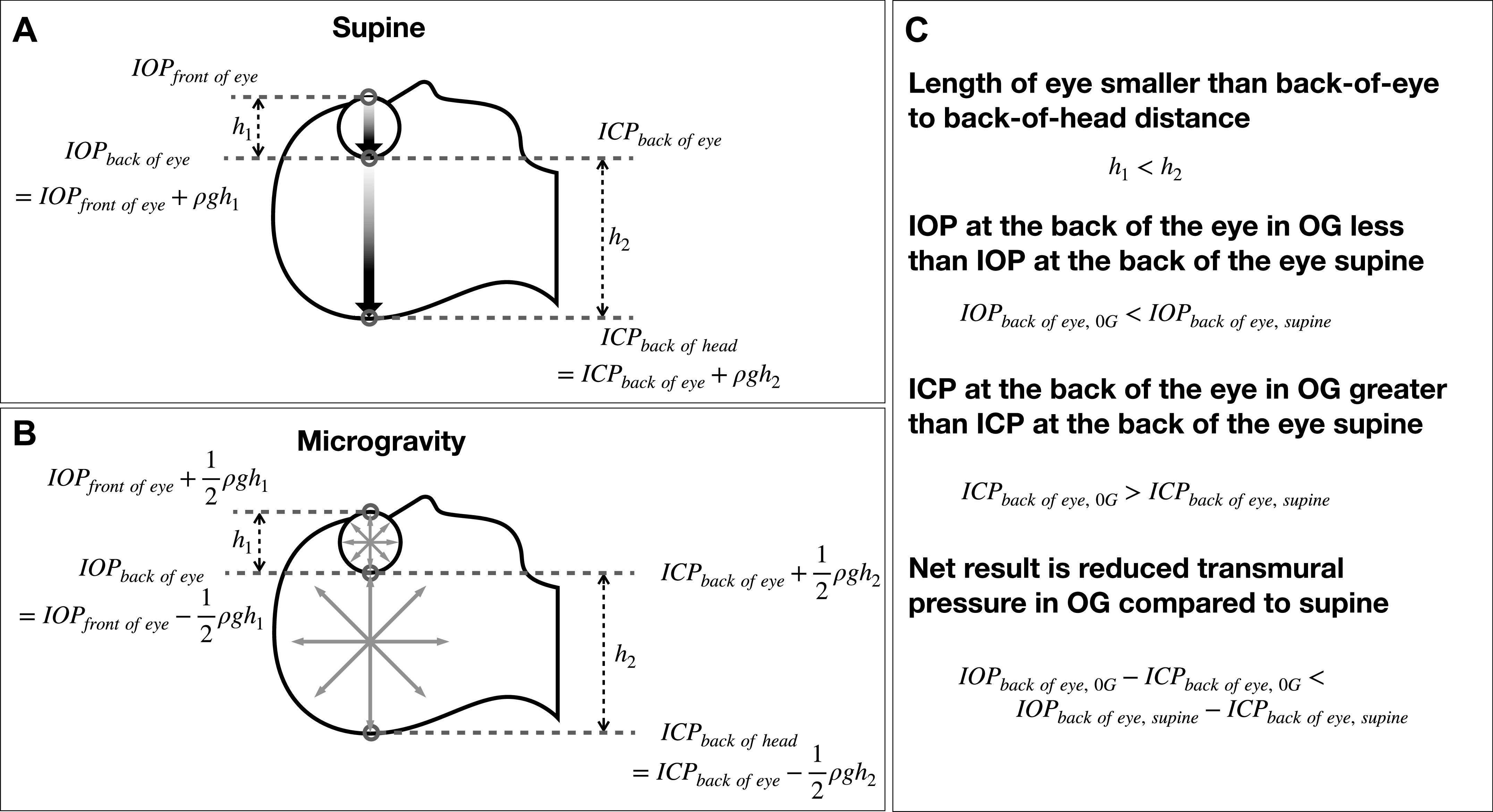
*A*: hydrostatic gradients increase IOP at the back of the eye but decrease ICP at the back of the eye. *B*: in weightlessness, removal of hydrostatic gradients reduces IOP at the back of eye while increasing ICP there. *C*: summary of the changes. ICP, intracranial pressure; IOP, intraocular pressure.

Figure 1*B* shows the possible situation when the eye and head are weightless. IOP becomes uniform throughout the eye, meaning IOP_back of eye_ is slightly reduced from what it was supine. ICP_back of eye_, however, increases. The combined outcome is that transmural pressure could be decreased (which shortens the eye) due to the removal of hydrostatic gradients alone. Even if the mean ICP and IOP in the center of the eye and the head are both decreased in weightlessness compared with the supine value, the transmural pressure could still be decreased. This could explain the paradox of increased SANS risk despite reduced venous pressures compared with supine.

A remaining question is why SANS would develop in those with higher preflight body weights. A potential answer comes from lower body negative pressure (LBNP) simulations with the numerical model (12). LBNP reduced head venous pressures, but this effect was not uniform. The IOP reduction with LBNP exceeded the ICP reduction leading to a change in the transmural gradient toward the development of SANS. This same effect may be happening when tissue weight is removed. Venous pressures in the head may be reduced when tissue weight is removed, but this effect could be greater on IOP than ICP leading to greater reductions in transmural pressure in higher-weight individuals.

This leads to the following potential hypothesis. In weightlessness, the removal of hydrostatic gradients reduces transmural pressure at the back of the eye. This effect is more pronounced in those who weigh more because they experience a larger reduction in venous pressure, and therefore greater reductions in transmural pressure, compared with those who weigh less. Also, since the usual postural and diurnal changes in CVP do not occur in space, these changes are persistent and unchanging. Over time, the persistently reduced transmural pressure across the back of the eye causes ocular remodeling and leads to the signs and symptoms of SANS.

This theory fits the available data and has practical implications. It incorporates the reduction in venous pressures in space as well as the observation that preflight body weight is associated with SANS. The modeling results also suggest that LBNP may not be a good countermeasure for SANS. It could reduce IOP more than ICP and lead to a worsening of the transmural pressure gradient across the back of the eye. Gx centrifugation, which recreates hydrostatic gradients, might be a countermeasure to study instead.

The theory has limitations. Figure 1 describes hydrostatic changes with an acute exposure to microgravity and does not incorporate time or the effect of long-term microgravity adaptations. In addition, recent data show that core body temperatures of astronauts are significantly increased, perhaps leading to different thermal expansion coefficients in different tissues, and this is not considered (15). Also, measuring pressures within the head to determine if the hydrostatic analysis is accurate is extremely difficult. The analysis of the hydrostatics within the eye and head and may be incorrect or oversimplified. The numerical model used for the analyses with body weight and LBNP includes numerous assumptions and may not be quantitatively accurate.

Nevertheless, this analysis strongly suggests that body weight and IOP are important to measure consistently in astronauts.

## GRANTS

The development of the numerical models was supported by grant CA03401 from the National Space Biomedical Research Institute through NCC 9–58 and by NASA EPSCoR Cooperative Agreement NNX13AD35A.

## DISCLOSURES

No conflicts of interest, financial or otherwise, are declared by the authors.

## AUTHOR CONTRIBUTIONS

J.C.B. conceived and designed research; J.C.B., M.L., S.D.P., V.A.-L., and A.M.F. analyzed data; J.C.B., M.L., S.D.P., V.A.-L., and A.M.F. interpreted results of experiments; J.C.B. and M.L. prepared figures; J.C.B. drafted manuscript; J.C.B., M.L., S.D.P., V.A.-L., and A.M.F. edited and revised manuscript; J.C.B., M.L., S.D.P., V.A.-L., and A.M.F. approved final version of manuscript.
